# Chronic pancreatitis of jejunal ectopic pancreas: a rare cause of chronic abdominal pain

**DOI:** 10.1093/jscr/rjae439

**Published:** 2024-07-03

**Authors:** Sonya Ann Smith, Jack Savage, Nicholas Roetger, Xavier Moar

**Affiliations:** Toowoomba Hospital, Pechey St, Toowoomba, Queensland 4350, Australia; University of Queensland, 308 Queen St, Brisbane, Queensland 4072, Australia; Toowoomba Hospital, Pechey St, Toowoomba, Queensland 4350, Australia; St Andrew’s Private Hospital, Radiology Department, 280 North St, Rockville, Queensland, 4350, Australia; Toowoomba Hospital, Pechey St, Toowoomba, Queensland 4350, Australia

**Keywords:** ectopic pancreas, chronic pancreatitis, diagnostic laparoscopy

## Abstract

Ectopic pancreas (EP) is an uncommon, congenital focus of pancreatic tissue that is discontinuous with the principal pancreas. A 62-year-old female underwent multiple investigations for chronic epigastric pain. EP was identified intra-operatively. On retrospection, earlier imaging showed a thickened segment of jejunum with inflammation of the surrounding small bowel mesentery, suggestive of jejunal EP pancreatitis. Histology confirmed ectopic pancreatic tissue, with sections of the EP showing evidence of previous acute and chronic pancreatitis. When no cause for chronic abdominal pain is found, diagnostic laparoscopy should be considered, and the small bowel inspected, to further investigate for rare causes of abdominal pain, such as EP.

## Introduction

Ectopic pancreatic rests, also known as heterotopic, aberrant, or ectopic pancreas (EP), are uncommon, congenital foci of pancreatic tissue that are discontinuous with the main pancreas. There are multiple theories postulating the development of EP. The misplacement theory suggests that a portion of pancreas separates from the main pancreas during foregut rotation [1, 2]. The metaplasia theory suggests that endodermal tissue develops into pancreatic tissue and migrates to the submucosa [1, 2]. The totipotent theory suggests that endodermal cells within the gastrointestinal tract differentiate into pancreatic tissue [1].

Research suggests the incidence of EP to be about 0.2% [3]. EP is found most often in the submucosa of the stomach, duodenum, and jejunum [4]. Macroscopically, these aberrant foci are usually spherical or oblong and 1–4 cm in size [5]. They are clearly demarcated from the surrounding small bowel and have a similar appearance to pancreatic tissue [5].

The literature suggests that EP is usually asymptomatic [4]. Significant variability exists in presentation amongst symptomatic cases, including abdominal pain, upper gastrointestinal bleeding, or dyspepsia [5]. Symptomatic EP tends to be larger than 1.5 cm and located in the stomach or duodenum [4]. EP is susceptible to the same pathologies that occur in the pancreas, such as pancreatitis and malignancy [6].

## Case report

A 62-year-old female was referred to clinic with 3 years of chronic abdominal pain. She described the pain as a constant discomfort in the epigastrium that radiated into the back. The pain was not worsened by fatty food or alcohol. There were no associated symptoms, such as vomiting or fevers.

Her surgical history is significant for an elective laparoscopic anterior resection for sigmoid diverticulitis 5 years prior, a hysterectomy 15 years prior for atypical hyperplasia, and an open appendicectomy as a child. She did not have a history of pancreatitis. Her medical history is significant for hypothyroidism for which she takes thyroxine. She is a life-long non-smoker and on average drinks two alcoholic drinks per week.

Her abdominal pain was investigated. Blood tests, including full blood count, liver function tests, renal function, and lipase, were unremarkable. Ultrasound of her abdomen was unremarkable; specifically, the biliary system lacked gallstones or biliary sludge. Her latest computed tomography (CT) abdomen and pelvis with contrast a few years prior to her review in clinic was reported as unremarkable. Upper gastrointestinal endoscopy and colonoscopy were unremarkable. Because her ongoing pain was significantly affecting her quality of life, she consented to an elective diagnostic laparoscopy to further investigate her pain.

**Figure 1:**
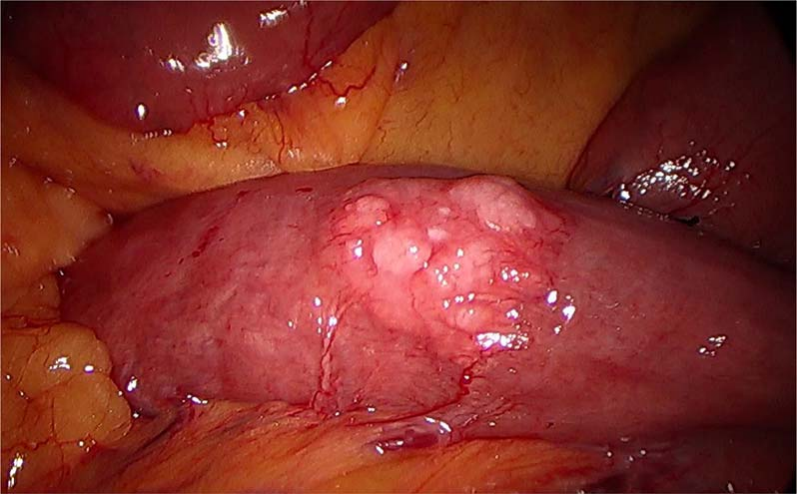
Intraoperative image during laparoscopy visualizing ectopic pancreas on serosa of the jejunum.

**Figure 2:**
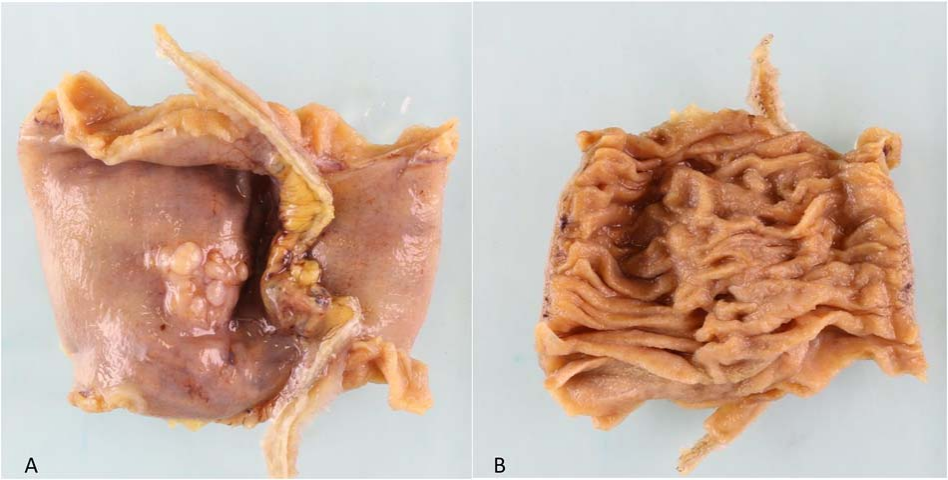
(a). Serosal surface of bowel. 16 mm white nodule noted on serosal surface. (b) Luminal surface of bowel. No abnormality seen macroscopically.

**Figure 3:**
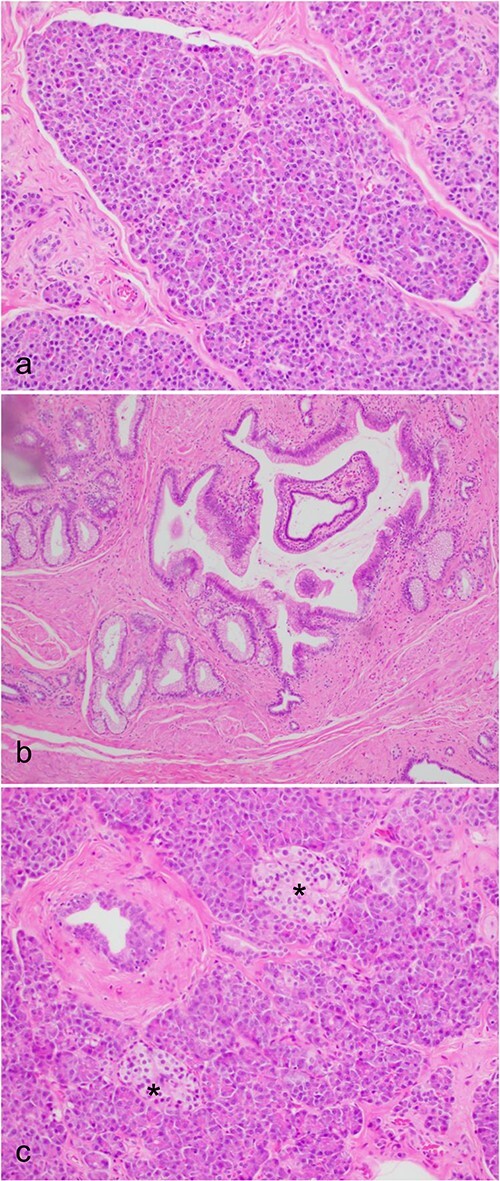
Microscopic histological images, showing (a) pancreatic acini and the exocrine pancreas within the ectopic pancreas. (b) The main pancreatic duct of the ectopic pancreas. (c) Islet’s of Langerhans (*) and the endocrine pancreas within the ectopic pancreas.

**Figure 4:**
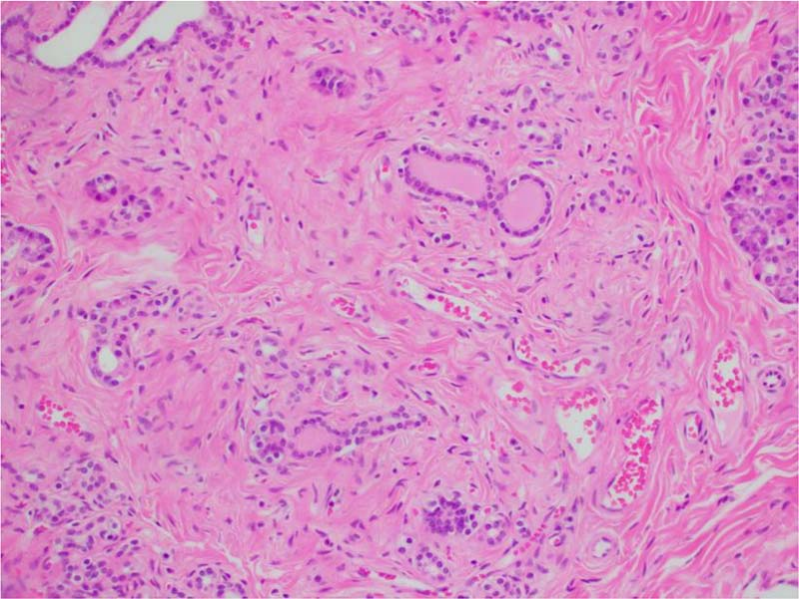
Microscopic histological image showing pancreatic atrophy and fibrosis with scattered chronic inflammation, indicative of past pancreatitis.

During a thorough inspection of the small bowel in its entirety, a yellow, lobulated oblong lump on the serosa of proximal jejunum was found (Fig. 1). A segment of small bowel including the lesion was resected and sent for histology. The small bowel was re-joined with a stapled anastomosis. Histopathological examination of the lesion revealed a 16 × 13 mm white, serosal nodularity (Fig. 1a). The underlying jejunal mucosa was normal (Fig. 2a). Histological sections were consistent with ectopic pancreatic tissue; they exhibited numerous acinar type glands associated with occasional islet of Langerhans, centred around a main pancreatic duct (Fig. 3). Some of the acinar units show gland drop out, fibrosis, atrophy, and scattered chronic inflammation, indicative of past and chronic pancreatitis (Fig. 4). Microscopically, the EP involved the submucosa and was abutting the serosa. No malignancy was present.

**Figure 5:**
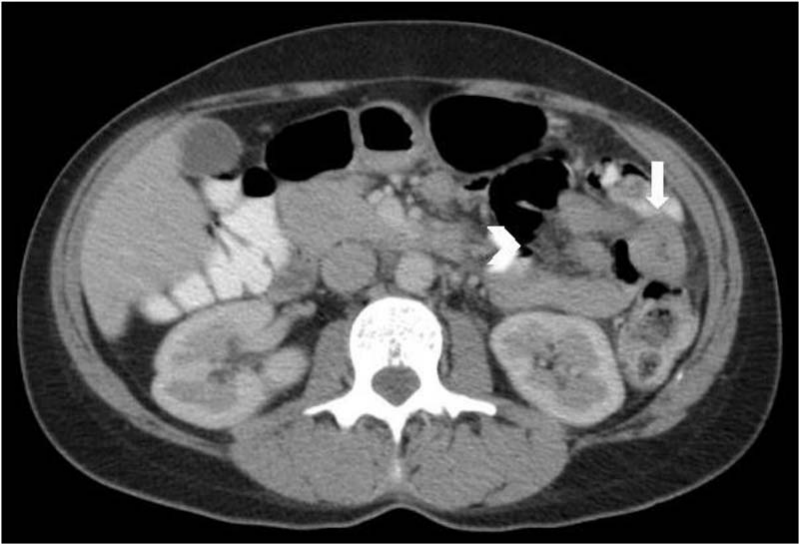
Axial CT abdomen and pelvis with portal venous phase and oral contrast, demonstrating a short segment of thickened jejunum (arrow) in the left upper quadrant with associated mesenteric enhancement (arrow head).

On retrospective review of this patient’s clinical course, she had presented to the emergency department many times with abdominal pain. Lipase was never elevated. On one presentation, a few years prior to her laparoscopy, a CT revealed a short segment of thickened jejunum in the left upper quadrant with associated mesenteric enhancement (Fig. 5); this was reported as a non-specific finding. C-reactive protein at this time was mildly elevated.

The patient had an uneventful post-operative recovery. She remains well at home 6 months post-operatively, with no further episodes of pain.

## Discussion

Only a few cases exist in the literature of possible pancreatitis within EP. One case has been reported of a 9-year-old in India with abdominal pain who underwent a bowel resection for a Meckel’s diverticulitis that contained inflamed ectopic pancreatic tissue [7]. Another case from the Netherlands reports a 58-year-old who presented with abdominal pain and was found to have EP within the jejunal mesentery [8]. Another case describes acute pancreatitis of EP within the gallbladder; this was also only diagnosed on post-operative histology [9].

In the cases of EP presenting with pain, lipase and amylase levels are usually normal; this is thought to be due to the minute size of the lesions [10]. Our case reflected this finding with non-elevated lipase during the patient’s emergency presentations. EP is seldom diagnosed pre-operatively; in a few cases, EP has been found pre-operatively with CT and endoscopy [11, 12].

Evidence of pancreatitis within EP is rarely identified on pre-operative imaging [3, 13]. The small bowel thickening and mesenteric inflammation on CT in our case, along with the patient's symptoms and the histology findings of past acute and chronic pancreatitis, appear to represent an episode of EP pancreatitis.

Many researchers suggest that both symptomatic and asymptomatic EP should be resected for a few reasons. Firstly, although rare, malignant transformation of the ectopic pancreatic tissue can occur; secondly, EP may cause pain or other symptoms in the future [4]. In most symptomatic cases, patients’ symptoms resolved after resection of the EP [4, 6]. Our case reflects this finding as well.

## Conclusion

When no cause is found, diagnostic laparoscopy should be considered and the small bowel inspected to further investigate for rare causes of abdominal pain, such as EP. If found intra-operatively, surgeons should consider resection of EP.
